# Supplementary material for: Effects of Menstrual Health and Hygiene on School Absenteeism and Drop-Out among Adolescent Girls in Rural Gambia
Source: Int J Environ Res Public Health. 2022 Mar 11;19(6):3337. doi: 10.3390/ijerph19063337 (PMC8954348; doi:10.3390/ijerph19063337)
Supplement: Supplementary file 1 [file ijerph-19-03337-s001.zip › ijerph-1601249-supplementary.pdf]

Back

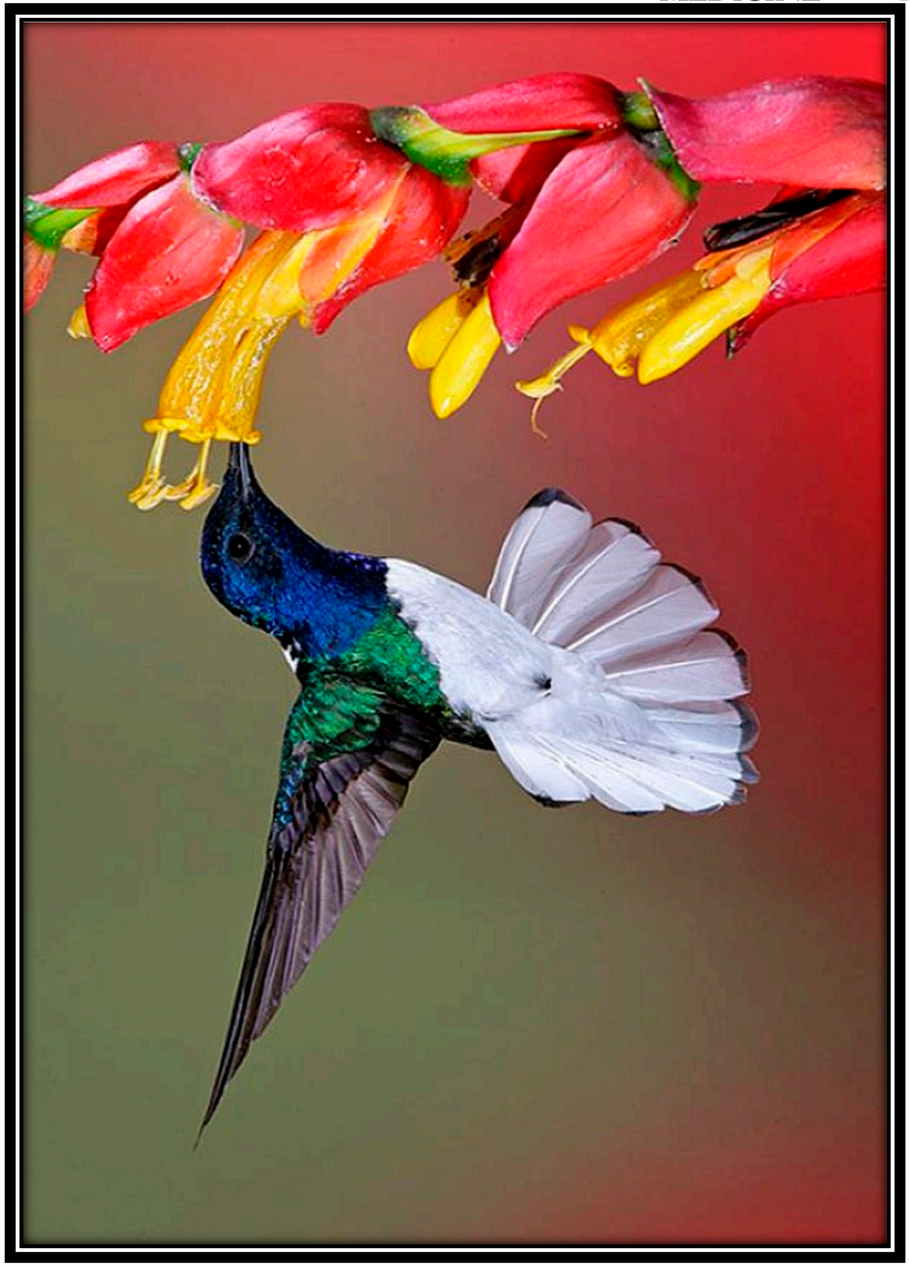

Participant ID: __I__I__I__I__I__I__I__I

Date: I__I__I/I__I__I/I__I__I__I__I

School:

Estimated date of menarche onset: I__I__I/I__I__I/I__I__I__I__I

Week 1		School					Period			
Day	Date	Yes	Half day	No	Holiday	Travelled	No	Light period	Moderate period	Heavy period
Sunday	29 Jan 17	☐	☐	☐	☐	☐	☐	☐	☐	☐
Monday	30 Jan 17	☐	☐	☐	☐	☐	☐	☐	☐	☐
Tuesday		☐	☐	☐	☐	☐	☐	☐	☐	☐
Wednesday		☐	☐	☐	☐	☐	☐	☐	☐	☐
Thursday		☐	☐	☐	☐	☐	☐	☐	☐	☐
Friday		☐	☐	☐	☐	☐	☐	☐	☐	☐
Saturday		☐	☐	☐	☐	☐	☐	☐	☐	☐

MRC The Gambia Unit

LONDON SCHOOL of HYGIENE & TROPICAL MEDICINE

Week 2		School					Period			
Day	Date	Yes	Half day	No	Holiday	Travelled	No	Light period	Moderate period	Heavy period
Sunday		☐	☐	☐	☐	☐	☐	☐	☐	☐
Monday		☐	☐	☐	☐	☐	☐	☐	☐	☐
Tuesday		☐	☐	☐	☐	☐	☐	☐	☐	☐
Wednesday		☐	☐	☐	☐	☐	☐	☐	☐	☐
Thursday		☐	☐	☐	☐	☐	☐	☐	☐	☐
Friday		☐	☐	☐	☐	☐	☐	☐	☐	☐
Saturday		☐	☐	☐	☐	☐	☐	☐	☐	☐

Week 3		School					Period			
Day	Date	Yes	Half day	No	Holiday	Travelled	No	Light period	Moderate period	Heavy period
Sunday										
Monday										
Tuesday										
Wednesday										
Thursday										
Friday										
Saturday										

Week 4		School					Period			
Day	Date	Yes	Half day	No	Holiday	Travelled	No	Light period	Moderate period	Heavy period
Sunday		☐	☐	☐	☐	☐	☐	☐	☐	☐
Monday		☐	☐	☐	☐	☐	☐	☐	☐	☐
Tuesday		☐	☐	☐	☐	☐	☐	☐	☐	☐
Wednesday		☐	☐	☐	☐	☐	☐	☐	☐	☐
Thursday		☐	☐	☐	☐	☐	☐	☐	☐	☐
Friday		☐	☐	☐	☐	☐	☐	☐	☐	☐
Saturday		☐	☐	☐	☐	☐	☐	☐	☐	☐

Week 5		School					Period			
Day	Date	Yes	Half day	No	Holiday	Travelled	No	Light period	Moderate period	Heavy period
Sunday		☐	☐	☐	☐	☐	☐	☐	☐	☐
Monday		☐	☐	☐	☐	☐	☐	☐	☐	☐
Tuesday		☐	☐	☐	☐	☐	☐	☐	☐	☐
Wednesday		☐	☐	☐	☐	☐	☐	☐	☐	☐
Thursday		☐	☐	☐	☐	☐	☐	☐	☐	☐
Friday		☐	☐	☐	☐	☐	☐	☐	☐	☐
Saturday		☐	☐	☐	☐	☐	☐	☐	☐	☐

Week 6		School					Period			
Day	Date	Yes	Half day	No	Holiday	Travelled	No	Light period	Moderate period	Heavy period
Sunday		☐	☐	☐	☐	☐	☐	☐	☐	☐
Monday		☐	☐	☐	☐	☐	☐	☐	☐	☐
Tuesday		☐	☐	☐	☐	☐	☐	☐	☐	☐
Wednesday		☐	☐	☐	☐	☐	☐	☐	☐	☐
Thursday		☐	☐	☐	☐	☐	☐	☐	☐	☐
Friday		☐	☐	☐	☐	☐	☐	☐	☐	☐
Saturday		☐	☐	☐	☐	☐	☐	☐	☐	☐

Week 7		School					Period			
Day	Date	Yes	Half day	No	Holiday	Travelled	No	Light period	Moderate period	Heavy period
Sunday		☐	☐	☐	☐	☐	☐	☐	☐	☐
Monday		☐	☐	☐	☐	☐	☐	☐	☐	☐
Tuesday		☐	☐	☐	☐	☐	☐	☐	☐	☐
Wednesday		☐	☐	☐	☐	☐	☐	☐	☐	☐
Thursday		☐	☐	☐	☐	☐	☐	☐	☐	☐
Friday		☐	☐	☐	☐	☐	☐	☐	☐	☐
Saturday		☐	☐	☐	☐	☐	☐	☐	☐	☐

MRC The Gambia Unit

LONDON SCHOOL of HYGIENE &TROPICAL MEDICINE

Week 8		School					Period			
Day	Date	Yes	Half day	No	Holiday	Travelled	No	Light period	Moderate period	Heavy period
Sunday		☐	☐	☐	☐	☐	☐	☐	☐	☐
Monday		☐	☐	☐	☐	☐	☐	☐	☐	☐
Tuesday		☐	☐	☐	☐	☐	☐	☐	☐	☐
Wednesday		☐	☐	☐	☐	☐	☐	☐	☐	☐
Thursday		☐	☐	☐	☐	☐	☐	☐	☐	☐
Friday		☐	☐	☐	☐	☐	☐	☐	☐	☐
Saturday		☐	☐	☐	☐	☐	☐	☐	☐	☐

February 2017

Sunday	Monday	Tuesday	Wednesday	Thursday	Friday	Saturday
			1	2	3	4
5	6	7	8	9	10	11
12	13	14	15	16	17	18
19	20	21	22	23	24	25
26	27	28				

March 2017

Sunday	Monday	Tuesday	Wednesday	Thursday	Friday	Saturday
			1	2	3	4
5	6	7	8	9	10	11
12	13	14	15	16	17	18
19	20	21	22	23	24	25
26	27	28	29	30	31	

April 2017

Sunday	Monday	Tuesday	Wednesday	Thursday	Friday	Saturday
						1
2	3	4	5	6	7	8
9	10	11	12	13	14	15
16	17	18	19	20	21	22
23	24	25	26	27	28	29
30						

May 2017

Sunday	Monday	Tuesday	Wednesday	Thursday	Friday	Saturday
	1	2	3	4	5	6
7	8	9	10	11	12	13
14	15	16	17	18	19	20
21	22	23	24	25	26	27
28	29	30	31			
